# The capillary pressure vs. saturation curve for a fractured rock mass: fracture and matrix contributions

**DOI:** 10.1038/s41598-023-38737-y

**Published:** 2023-07-25

**Authors:** Alejandro Cardona, Qi Liu, J. Carlos Santamarina

**Affiliations:** 1 Institute for Geophysics, Jackson School of Geosciences, The University of Texas at Austin, 10601 Exploration Way, Austin, TX 78758 USA; 2 King Abdullah University of Science and Technology KAUST, Thuwal, 23955-6900 Saudi Arabia; 3 School of Civil and Environmental Engineering, Georgia Institute of Technology, 790 Atlantic Drive, N.W., Atlanta, GA 30332 USA

**Keywords:** Carbon capture and storage, Hydrology, Fluid dynamics, Civil engineering

## Abstract

The fractal topography of fracture surfaces challenges the upscaling of laboratory test results to the field scale, therefore the study of rock masses often requires numerical experimentation. We generate digital fracture analogues and model invasion percolation to investigate the capillarity-saturation *P*_*c*_*-S*_*w*_ fracture response to changes in boundary conditions. Results show that aperture is Gaussian-distributed and the coefficient of variation is scale-independent. The aperture contraction during normal stress increments causes higher capillary pressures and steeper *P*_*c*_*-S*_*w*_ curves, while shear displacement results in invasion anisotropy. The three-parameter van Genutchen model adequately fits the fracture capillary response in all cases; the capillary entry value decreases with fracture size, yet the fracture *P*_*c*_*-S*_*w*_ curve normalized by the entry value is size-independent. Finally, we combine the fracture and matrix response to infer the rock mass response. Fracture spacing, aperture statistics and matrix porosity determine the rock mass capillarity-saturation *P*_*c*_*-S*_*w*_ curve. Fractures without gouge control the entry pressure whereas the matrix regulates the residual saturation at high capillary pressure *P*_*c*_.

## Introduction

Mixed fluid conditions are common in fractured rocks and affect both engineering applications and natural processes^1,2^. Examples include energy resource extraction such as hydrocarbons and geothermal^3,4^, the vadose zone and environmental remediation^5,6^, geological storage of CO_2_ and nuclear waste^7,8^, and hydrothermal alteration and ore deposition^9^.

In the absence of gouge, fractures provide preferential flow pathways that dominate fluid flow. Then, invasion can readily bypass wetting (and even non-wetting) fluids in the matrix and fast injection studies tend to conclude low displacement efficiency (e.g., CO_2_ injection due to viscous fingering, and aggravated by fractures and heterogeneity). On the other hand, long-term analyses must be based on capillarity-saturation curves at thermodynamic equilibrium.

Pore size distribution, pore interconnectivity and spatial variability determine the relationship between the capillary pressure and the degree of saturation at equilibrium *P*_*c*_-*S*_*w*_^10^. These constitutive equations capture the entry pressure and the sensitivity of saturation to changes in capillary pressure. Most numerical codes rely on these expressions to analyze coupled processes^11,12^.

Measurement techniques for the intact rock *matrix* are well established and rely on porous plates and tensiometers^13^, mercury injection^14^, centrifuge methods^15^, controlled suction^16,17^, dew point hydrometer^18^, and thermal/electrical conductivity measurements on calibrated porous stones^19^. The measured capillary pressure–saturation curves are relatively smooth for intact rocks and can be fitted with simple functions^20,21^.

In general, we can anticipate that the geometric aperture and its spatial correlation control capillary phenomena in *fractures*^22,23^. However, stress-sensitive aperture^24–26^, fractal fracture topography^27^, specimen size limitations and fracture-matrix interaction hinder the experimental determination of *P*_*c*_*-S*_*w*_ curves in fractures and fractured rocks^28–30^. Furthermore, *P*_*c*_-*S*_*w*_ curves would take weeks to months to reach thermodynamic equilibrium, particularly when trapped zones equilibrate through dissolution-diffusion processes^31,32^; in fact, most published laboratory studies report rapid fluid invasion tests^33^.

In view of spatial and temporal scale limitations, numerical experimentation emerges as a necessary tool to study mixed fluid conditions in fractured rocks under long-term thermodynamic equilibrium. Previous numerical studies have explored the link between the fracture aperture and capillary pressure^34–36^, and their associated changes with normal stress^37,38^. Yet, realistic fracture surface generation algorithms, the evolution of aperture with normal stress and shear displacement, and implications on the capillarity of fractured rock masses require further research.

This study explores the relationship between capillary pressure and saturation in fractured rocks, with emphasis on fracture-matrix interaction *at equilibrium* following the invasion of a non-wetting phase (Note: fast immiscible fluid invasion and transient saturation are not part of this study).

## Fracture studies: methodology

First, we investigate the effects of normal stress, shear displacement and fracture surface topography on the fracture capillary response. We generate digital fracture analogues that resemble natural fractures and bring the two rough surfaces together to create the fracture pore space. Then, we use network model simulations to investigate the effects of surface roughness and matedness on the fracture capillarity-saturation curve, and its evolution during normal loading and shear displacement. Fracture generation, response to normal stress and shear displacement, and fluid invasion modeling are described next.

### Fracture generation

**Figure 1:**
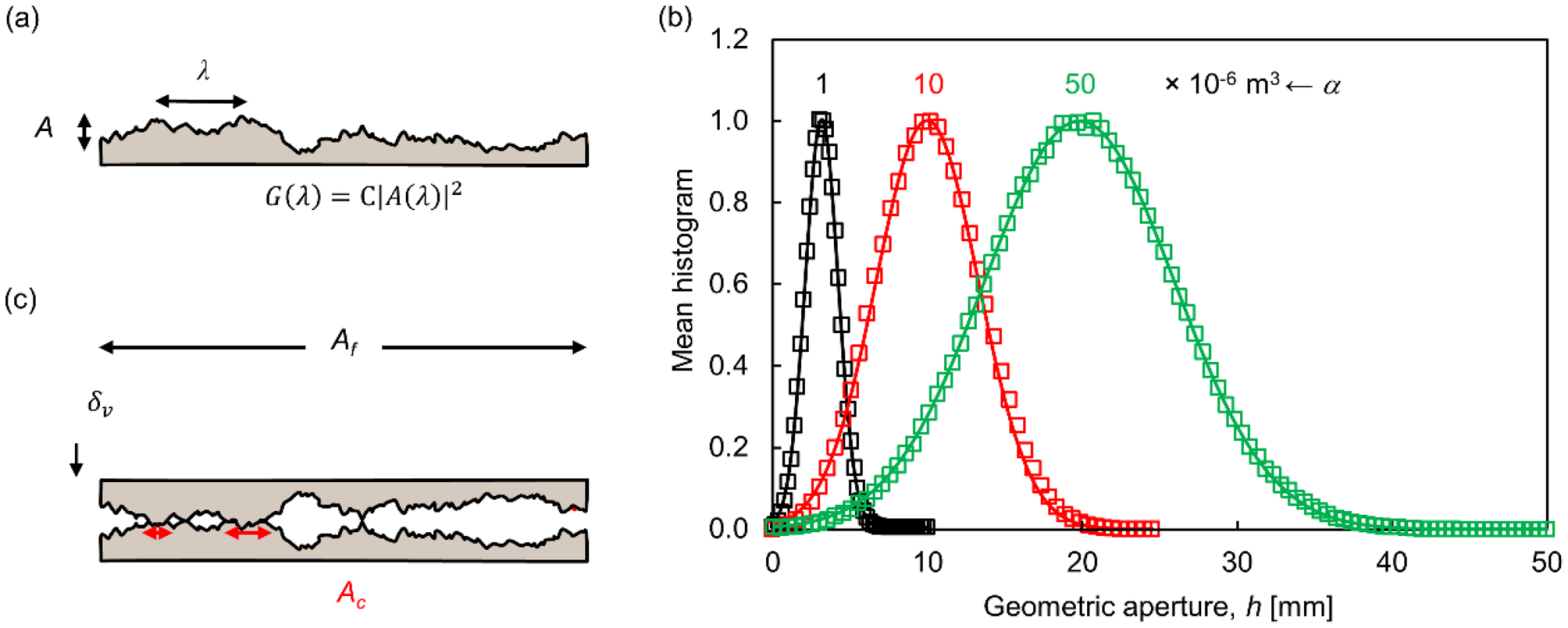
Fracture generation and deformation model. (**a**) Surface roughness (*α* = 1 × 10^−6^ m^3^_,_ *β* = 2.9. Vertical cross-section of a 3D fracture; scale exaggerated by a factor of ~ 2). (**b**) Geometric aperture distribution for “unmated” 3D fracture surfaces. Mean trends based on 100 realizations (markers: computed values; solid lines: fitted normal distribution). Black: *α* = 1 × 10^–6^ m^3^_,_ *β* = 2.9, *μ*_*h*_ = 3.2 mm, *s*_*h*_ = 1 mm; red: *β* = 2.9, *α* = 1 × 10^–5^ m^3^_,_ *μ*_*h*_ = 10 mm, *s*_*h*_ = 3.3 mm; green: *α* = 5 × 10^–5^ m^3^_,_ *β* = 2.9, *μ*_*h*_ = 19.8 mm, *s*_*h*_ = 6 mm. Fracture size: 0.1 × 0.1 m, cell size: 0.1 mm. (**c**) Contact model: *A*_*c*_ = *A*_*f*_ (*σ*/*σ*_*y*_).

The power spectral density of rock fracture surfaces *G*(*λ*) [m^3^] follows a power law with respect to wavelength *λ* [m]^27,39,40^ (Fig. 1a):

1 $$G\left(\lambda \right)=\alpha {\left(\frac{\lambda }{{\lambda }_{ref}}\right)}^{\beta },$$

where the reference wavelength is ${\lambda }_{ref}=1 \mathrm{m}$. This power equation implies a fractal topography and provides a convenient framework for numerical studies. The wave amplitude* X*(*λ*) [m] for a certain wavelength *λ* is related to the spectral density as *G*(*λ*) = *C* |*X*(*λ*)|^2^, where the scaling factor *C* = *N∆x/4* [m] depends on the selected Fourier pair and transform definition (i.e., one-sided vs. two-sided). We use the Inverse Fast Fourier Transform to synthesize the surface roughness profile in space by assuming a uniformly distributed random phase (see similar surface generation models in Refs.^38,41^). The fractal topography implies lack of a characteristic scale or representative equivalent size. However, fractal geometries have limits in natural systems; in our case, the longest wavelength is the fracture size under consideration.

Two contiguous rock surfaces define a fracture. We capture matedness by considering the correlation between them. There are two end-members: “perfectly mated fractures” consist of two identical surfaces with opposite orientations, and “unmated fractures” which are made of two uncorrelated surfaces. Figure 1b shows the aperture size distribution of unmated fracture surfaces generated using various *α*-factors (Eq. (1)).

### Fracture deformation

#### Deformation due to normal stress

We adopt a simple rigid-plastic contact model to compute the normal deformation due to a normal stress *σ* [MPa]. The rock reaches its yield stress *σ*_*y*_ [MPa] at the true fracture contact area *A*_*c*_ [m^2^]. Then, equilibrium implies,

2 $${A}_{c}\left(\sigma \right)={A}_{f}\frac{\sigma }{{\sigma }_{y}},$$

where *A*_*f*_ [m^2^] is the total fracture area. The two fracture surfaces interpenetrate a vertical distance *δ*_*v*_ to satisfy the computed contact area *A*_*c*_ (Eq. (2)—see Fig. 1c). While mass is not conserved at contacts, its effect on aperture distribution is negligible due to the small contact area *A*_*c*_. The selected rigid-plastic model depends on a single variable -the yield stress *σ*_*y*_- in line with Ockham’s principle of parsimony^42^. For comparison, the simplest elastoplastic model with post-peak softening requires ≥ 4 variables^38,43^ and additional parameters would be needed to track the evolution of the yield strength with contact deformation^44^. The rigid-plastic model is simple to implement, supports robust analyses and is adequate for capillary studies.

#### Shear displacement

We impose shear by displacing the two surfaces as rigid bodies without changing their surface topographies; natural processes are more complex and involve asperity shearing and/or overriding. The periodic boundary condition maintains a constant fracture area *A*_*f*_ during shear displacement whereby the moving surface re-appears on the opposite side. This boundary condition assumes the shear displacement is a subset of an infinite medium.

### Invasion percolation for drainage

After shear displacement and normal loading, we map the resulting *N* × *N* aperture field $\it {\text{h}}_{i,j}$ with spatial resolution Δ*x* = Δ*y* onto a *N*_*p*_ × *N*_*p*_ square lattice with fourfold connectivity where *N*_*p*_ < *N* (i.e., a “checkerboard” model—see Refs.^45–47^). The height *h*_*avg*_ of each cell is the average aperture in the original fracture within the corresponding area Δ*l* × Δ*l*, so that $\sum {h}_{i,j}\Delta {x}^{2}\approx {h}_{avg}\Delta {l}^{2}$.

The selected cell size Δ*l* × Δ*l* defines the aperture resolution. While the pressure-dependent saturation of a given pore depends on the pore geometry, the global trends exhibit limited sensitivity to local details: results from a focused numerical study reveal that aperture averaging in Δ*l* × Δ*l* affects only the lower end of the capillary-saturation *P*_*c*_-*S*_*w*_ curve, i.e., at high capillary pressures and low degrees of saturation. Furthermore, apertures *h* are much smaller than wavelengths in natural fractures (*h*/*λ* <  < 1—Ref.^27^); therefore, the in-plane radius of curvature is negligible and capillarity-saturation results are unaffected by aperture averaging when Δ*l* < 10Δ*x*. Previous studies show that the in-plane curvature can influence invasion patterns^34,38,48^; however, our fracture generation model ensures *h*/*λ* <  < 1 and the capillary pressure is only a function of the aperture.

**Figure 2:**
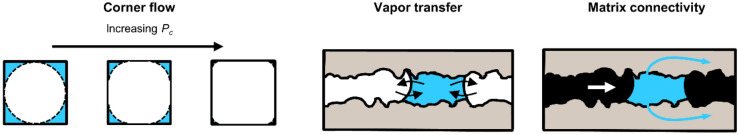
Processes involved in preventing the trapping of the wetting phase in fractures.

The invasion percolation algorithm assumes equilibrium at any given pressure (i.e., neither viscous forces nor time effects), and non-trapping of the wetting phase. Various pore-scale phenomena justify the non-trapping assumption, including (Fig. 2): corner flow along rough surfaces^31^, fluid transport into the matrix^49,50^, water evaporation and vapor pressure equilibration^51^. While these processes have characteristic time scales, the equilibrium assumption disregards any transient trapping of the wetting phase.

We implement the Young–Laplace equation in terms of the aperture-induced curvature, *P*_*c*_ = *T*_*s*_/*h* (Note: this expression assumes cylindrical interfaces given that *h* < Δ*l* and a perfectly wetting mineral surface *θ* = 0°). The largest aperture connected to the inlet is invaded first and defines the entry value. All apertures connected to the non-wetting phase throughout the medium are candidates for invasion. Invasion proceeds by monotonically increasing the capillary pressure to define the capillary pressure versus saturation *P*_*c*_-*S*_*w*_ curve.

## Results

### The fracture *P*_*c*_-*S*_*w*_ curve

**Figure 3:**
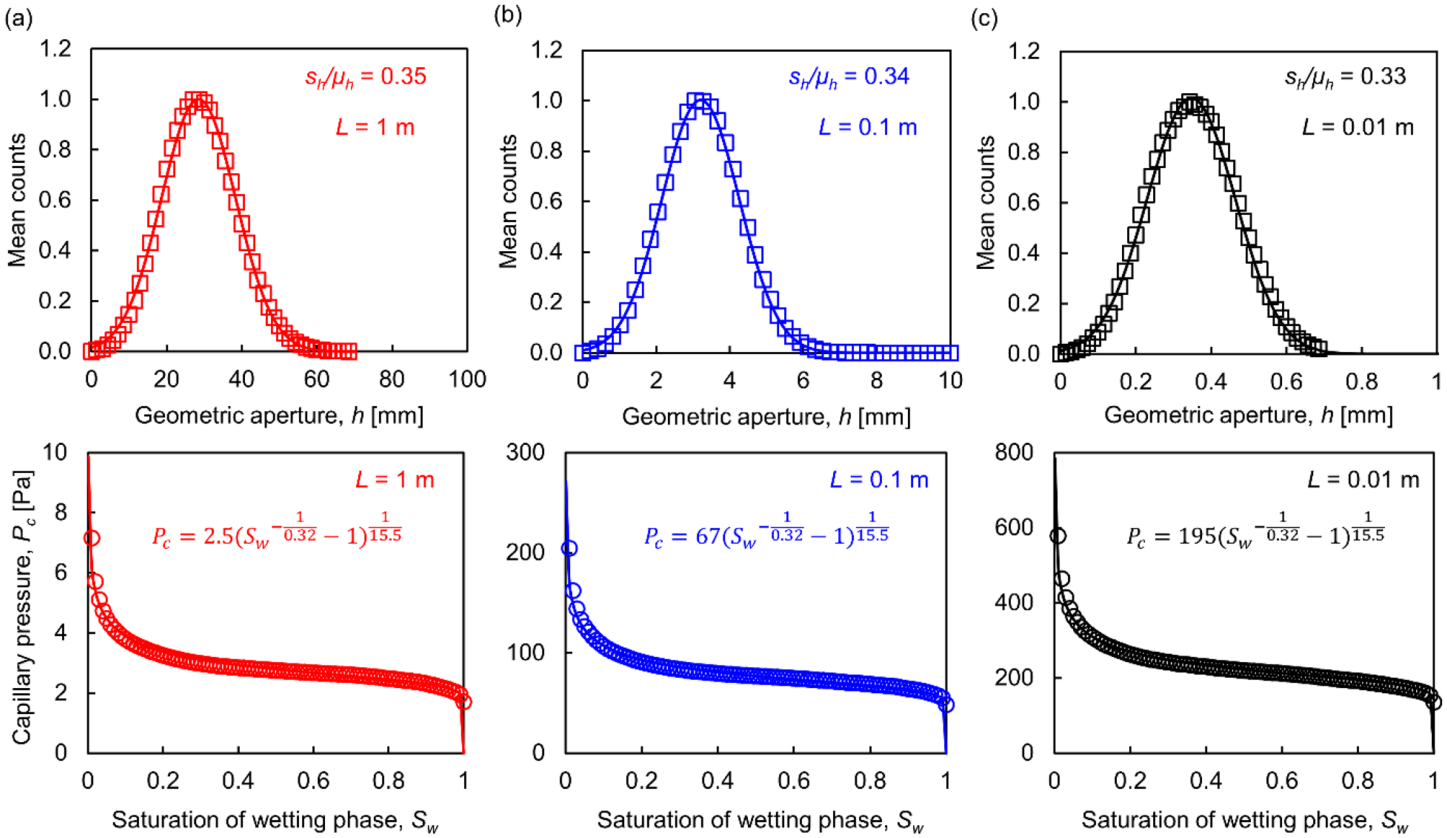
Aperture size distribution and *P*_*c*_*-S*_*w*_ curves for uncorrelated fractures of size *L*x*L*. (**a**) *L* = 1 m. (**b**) *L* = 0.1 m. (**c**) *L* = 0.01 m. The number of cells is *N*_*p*_ × *N*_*p*_ = 100 × 100 in all cases, therefore, the cell size *Δl* decreases proportionally to fracture size. Unmated fractures generated with power spectral density parameters *α* = 1 × 10^–6^ m^3^ and *β* = 2.9 (Eq. (1)). Numerical results shown as empty markers are the mean values of 100 numerical realizations and are fitted with a Gaussian distribution (geometric aperture) and the van Genuchten model (capillary pressure).

Fractures vary over a wide range of length scales. Figure 3 shows the aperture size distribution for three uncorrelated and unmated fractures of size *L*_*x*_ × *L*_*y*_ = 1 × 1 m, 0.1 × 0.1 m and 0.01 × 0.01 m subjected to zero normal stress (power spectral density parameters: *α* = 1 × 10^–6^ m^3^, *β* = 2.9). The selected fracture variables represent natural conditions and highlight size effects. Each curve represents an average of 100 numerical realizations in order to obtain statistically representative results. The mean aperture size distribution is Gaussian-distributed in the three cases, with almost the same coefficient of variation *s*_*h*_/*μ*_*h*_ defined in terms of the aperture standard deviation *s*_*h*_ and the mean aperture *μ*_*h*_ (see Fig. 3).

The capillary pressure *P*_*c*_ at a given saturation *S*_*w*_ increases as the fracture size decreases due to inverse relationship between aperture and fracture size. We fit the mean *P*_*c*_-*S*_*w*_ curve using the three-parameter van Genutchen model^52^:

3 $${P}_{c}={P}_{0}{\left({{S}_{w}}^{-1/{m}_{1}}-1\right)}^{1/{m}_{2}},$$

where *P*_*0*_ [Pa] relates to the entry value, and *m*_*1*_ and *m*_*2*_ capture the sensitivity of saturation to capillary pressure. This three-parameter model provides an excellent fit compared to the two-parameter van Genutchen and Brooks-Corey models. While the *P*_*0*_ value is inversely proportional to the fracture size *L*, the *m*_*1*_ and *m*_*2*_ parameters remain constant regardless of the fracture size for surfaces generated with the same power spectral density *α* and *β* parameters (Eq. (1)); hence, the normalized (*P*_*c*_*/P*_*0*_) − *S*_*w*_ curves are scale-independent.

#### The effect of normal stress

**Figure 4:**
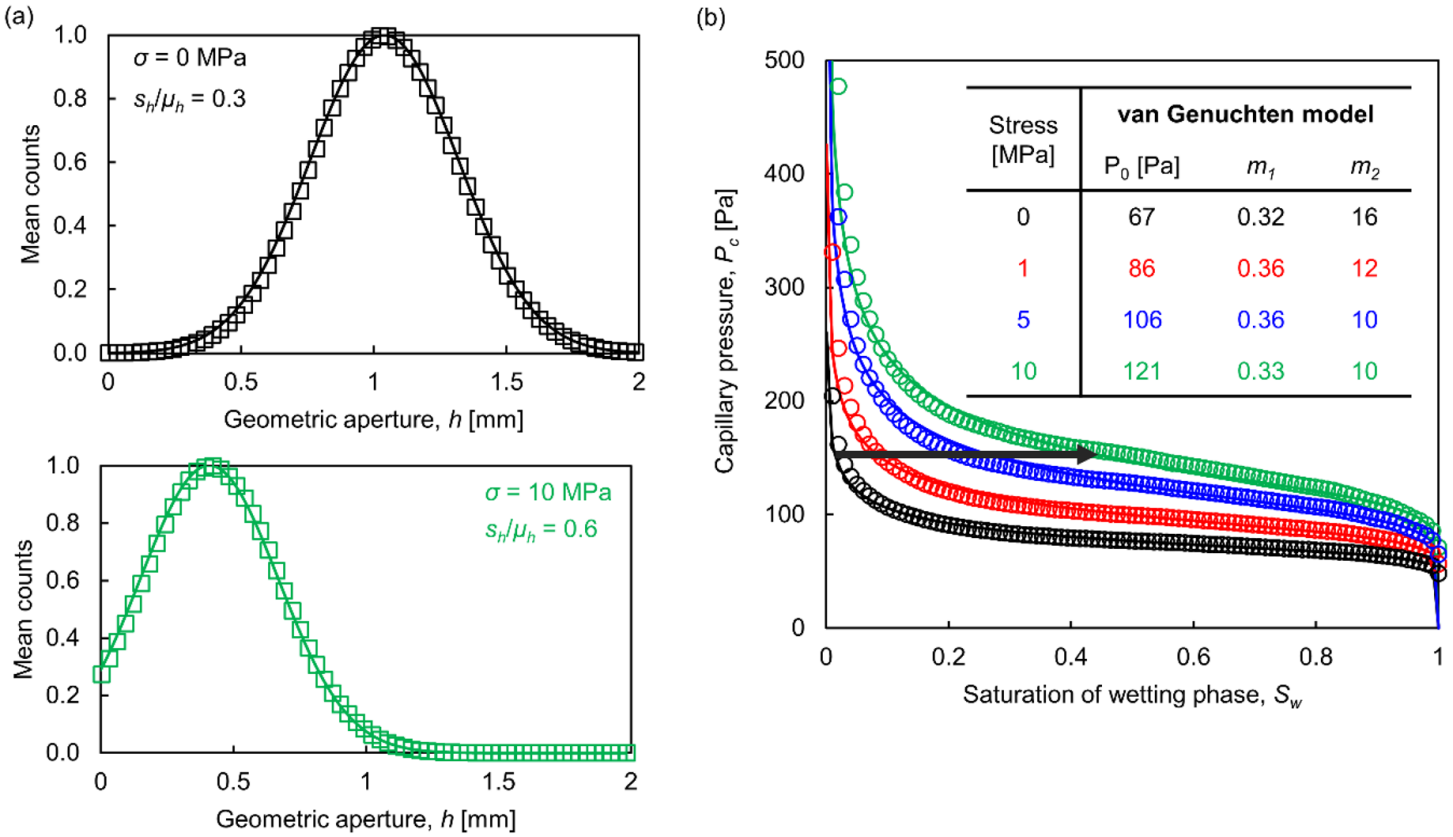
Evolution of fracture aperture and *P*_*c*_-*S*_*w*_ curve with normal stress. (**a**) Aperture size distribution at normal stress *σ* = 0 and *σ* = 10 MPa. Numerical results shown as empty markers are the mean values of 100 numerical realizations and are fitted with truncated Gaussian distribution (model parameters: *α* = 1 × 10^−7^ m^3^_,_ *β* = 2.9, *L* × *L* = 0.1 × 0.1 m, *Δl* = 1 mm, *σ*_*y*_ = 200 MPa). (**b**)* P*_*c*_*-S*_*w*_ curve for unmated fractures subjected to normal stress. The van Genuchten model (solid line) is used to fit numerical results (empty markers: mean of 100 numerical realizations). The arrow indicates the saturation path for a fracture that experiences an increase in normal stress at constant capillary pressure.

As the normal stress increases, the aperture size distribution shifts towards smaller values and a cutoff at zero aperture emerges as contact yield results in zero aperture contact points (Fig. 4a). The coefficient of variation of the fitted truncated Gaussian distributions increases with normal stress, and the aperture field exhibits higher variability.

Reduced apertures require higher capillary pressures for the same degree of saturation and the slope of the *P*_*c*_*-S*_*w*_ curve increases (Fig. 4b). The evolution of the van Genutchen model parameters *P*_*0*_, *m*_*1*_ and *m*_*2*_ are shown in the inset (Fig. 4b). Note that higher normal stress and fracture closure increase the degree of saturation of the wetting phase at constant capillary pressure (see arrow in Fig. 4b).

#### The effect of shear displacement

The shearing of unmated, uncorrelated fracture surfaces results in statistically identical aperture fields and the capillarity-saturation *P*_*c*_-*S*_*w*_ trends remain the same (in the absence of asperity shearing and gouge formation).

**Figure 5:**
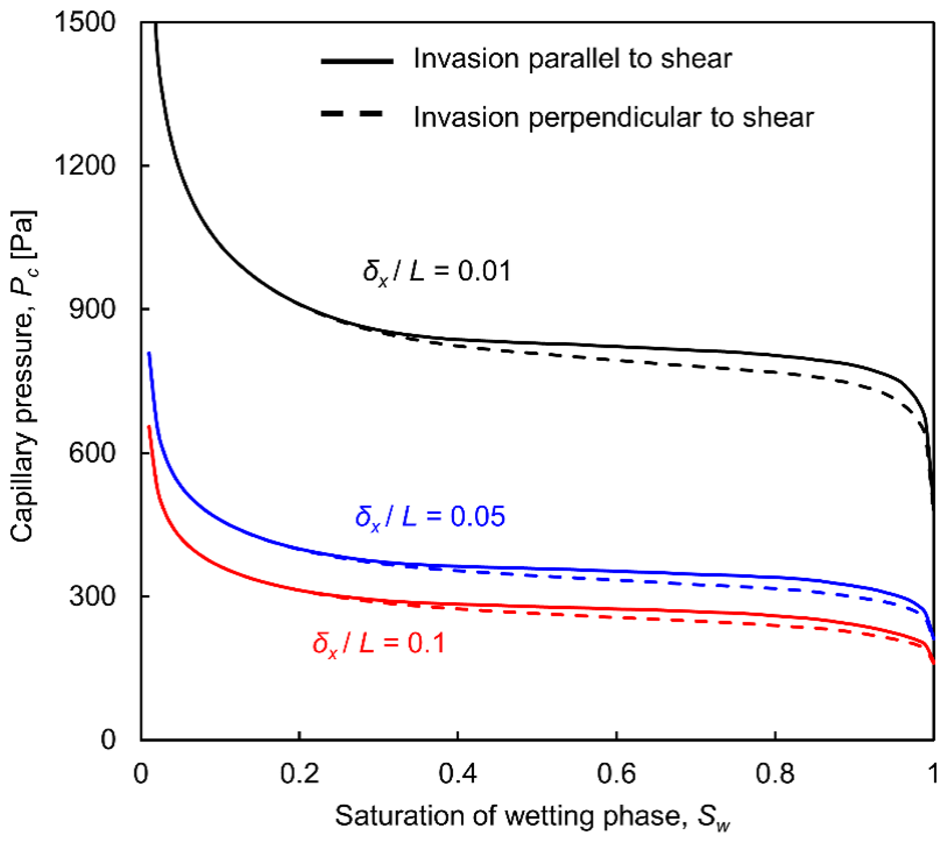
Capillary pressure vs. saturation *P*_*c*_*-S*_*w*_ curves for initially mated fractures that experience shear displacement at zero normal stress. Shear displacement occurs along the *x*-direction and is normalized with respect to the fracture sizes *L.* Plotted results are the mean values of 100 realizations. Model parameters: *α* = 1 × 10^−6^ m^3^, *β* = 2.9, *L* × *L* = 0.01 × 0.01 m, *Δl* = 0.1 mm.

Shear displacement causes aperture changes only when there is some initial degree of matedness between surfaces^27^. Figure 5 shows the capillarity-saturation *P*_*c*_-*S*_*w*_ curves for initially mated fractures: as the shear displacement increases, the mean aperture increases and the capillary pressure decreases for a given degree of saturation.

Shear displacement induces anisotropy in the aperture field of initially mated fractures as aperture ridges emerge transverse to the shear direction. Results in Fig. 5 show that the ensuing anisotropy in aperture connectivity produces slightly different *P*_*c*_-*S*_*w*_ curves -particularly at low *P*_*c*_ values- when the capillary pressure is controlled at a boundary that is normal or parallel to ridges.

### The *P*_*c*_-*S*_*w*_ curve for the fractured rock mass

**Figure 6:**
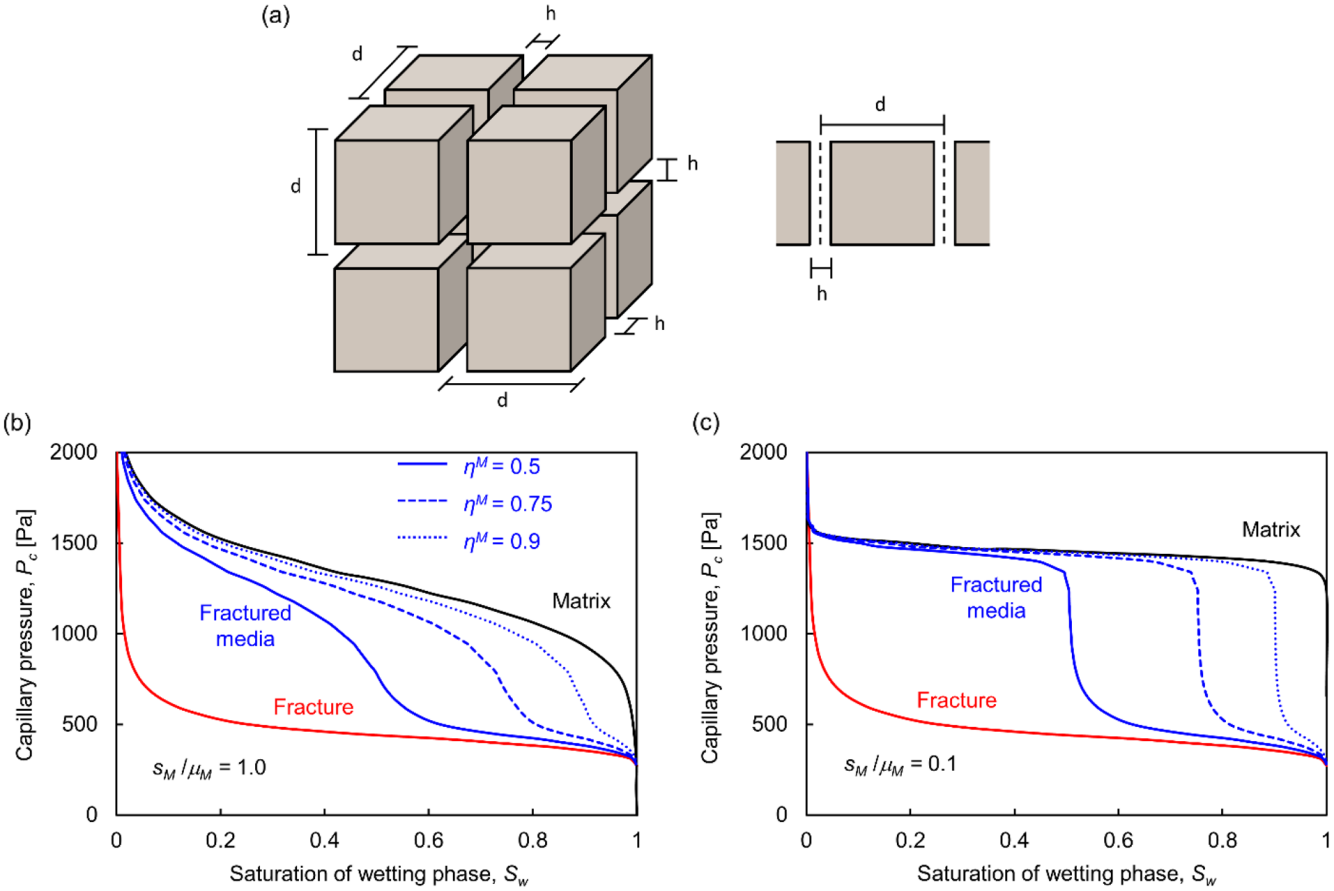
Fractured rock mass capillary behavior. (**a**) The fractured rock mass with three orthogonal fracture sets with the same spacing *d* in the three directions. (**b,c**) *P*_*c*_-*S*_*w*_ curve for the fractured medium with a (**b**) heterogeneous (*s*_*M*_/*μ*_*M*_ = 1.0) and (**c**) homogeneous (*s*_*M*_/*μ*_*M*_ = 0.1) rock matrix lognormal pore size distribution (see Ref.^53^ for rock matrix pore network model). Model parameters used to compute the fracture *P*_*c*_-*S*_*w*_ curve: *α* = 1 × 10^–7^ m^3^_,_ *β* = 2.9, *L* × *L* = 0.1 × 0.1 m, *Δl* = 1 mm. Model parameters for the matrix: *μ*_*M*_ = 0.1 mm, *L* × *L* = 0.1 × 0.1 m, *Δl* = 1 mm.

The fracture and matrix *P*_*c*_*-S*_*w*_ curves combine to define the capillary response of the fractured rock mass. Consider three orthogonal fracture sets with the same spacing *d* in the three directions so that the repetitive unit is of size ${d}^{3}$ (Fig. 6a). The matrix porosity *n*, the mean aperture size *μ*_*h*_ and the fracture spacing *d* determine the volume of voids in the matrix ${V}_{v}^{M}\approx n{d}^{3}$ and in fractures ${V}_{v}^{F}\approx 3{d}^{3}\left({\mu }_{h}/d\right)$ for ${\mu }_{h}/d\ll 1$. The volume of voids in the matrix ${V}_{v}^{M}$ becomes a significant fraction of the total volume of voids ${V}_{v}^{M}$+${V}_{v}^{F}$ for small aperture to spacing ratios *μ*_*h*_*/d* and high matrix porosity *n*:

4 $${\eta }^{M}=\frac{{{V}_{v}}^{M}}{{{V}_{v}}^{M}+{{V}_{v}}^{F}}\approx \frac{n}{n+3\left(\frac{{\mu }_{h}}{d}\right)}.$$

Conversely, the storativity in fractures gains relevance in rock masses with low matrix porosity *n*

5 $${\eta }^{F}=\frac{{{V}_{v}}^{F}}{{{V}_{v}}^{M}+{{V}_{v}}^{F}}\approx \frac{3\left(\frac{{\mu }_{h}}{d}\right)}{n+3\left(\frac{{\mu }_{h}}{d}\right)},$$

where *η*^*F*^ = *1* − *η*^*M*^. The fracture and the matrix share the same capillary pressure at equilibrium, therefore, the resultant *P*_*c*_ − *S*_*w*_ curve is a void-volume average of the saturation contributed by the fracture and the matrix *S*_*w*_^*RM*^ = *S*_*w*_^*F*^(*1* − *η*^*M*^) + *S*_*w*_^*M*^*η*^*M*^.

Let’s consider the matrix and the fracture *P*_*c*_*-S*_*w*_ curves and combine them for various matrix void fractions *η*^*M*^ to estimate the rock mass capillary response. We compute the fracture *P*_*c*_*-S*_*w*_ curve using the algorithm described above, and a similar algorithm for the matrix where pores are represented as connected tubes with capillary pressure *P*_*c*_ = 2*T*_*s*_/*r* (see model details in Ref.^53^). Results in Fig. 6 show that fractures -without gouge- control the entry pressure, whereas the matrix dominates the behavior at high capillary pressure. As the *η*^*M*^ fraction increases, the rock mass capillary-saturation curves approach the matrix *P*_*c*_*-S*_*w*_ curve.

## Discussion

The long-term saturation of a fractured rock mass will depend on the capillary pressure and the degree of saturation at equilibrium *P*_*c*_-*S*_*w*_*.* This will determine the original oil saturation profile in the reservoir and the residual oil after production, the long-term CO_2_ and H_2_ storage capacity, the distribution of LNAPLs and DNAPLs contaminants and environmental remediation strategies, the residual distribution of hydraulic fracturing fluids and the relative permeability of the resulting fractured rock mass.

The numerical study revealed surprising emergent properties. In particular, why is the aperture coefficient of variation *s*_*h*_/*μ*_*h*_ independent of fracture size? (Fig. 3). The fracture surface topography *z* is the sum of *k* independent sinusoids with amplitudes *a*_*i*_ that follow the power law in Eq. (1) and random phase *φ*_*i*_, $z={\sum }_{i}^{k}{z}_{j}={\sum }_{i}{a}_{i}\mathrm{sin}({\omega }_{i}{x}_{j}+{\varphi }_{i})$. The probability density function of the sum of random variables *f*_*z*_ is the convolution of the individual density functions, ${f}_{z}$ = ${f}_{{z}_{1}}$∗${f}_{{z}_{2}}$… ∗ ${f}_{{z}_{k}}$. In the limit *k* → ∞, the central limit theorem emerges and ${f}_{z}$ converges to a Gaussian distribution^54^. While the central limit theorem often requires ${f}_{{z}_{i}}$ to be independent and identically distributed, ${f}_{z}$ can converge to a Gaussian distribution for non-identical density functions (Lyapunov’s Central limit theorem—Ref.^55^). Then, the fracture surface topography *z* satisfies a Gaussian distribution, *z* ~ *N (μ*_*z*_*, s*_*z*_^2^).

The fracture geometric aperture is the subtraction of the top *z*_*t*_ and bottom *z*_*b*_ surface topographies, *h* = *z*_*t*_ − *z*_*b*,._ Therefore, the fracture aperture values also exhibit a Gaussian distribution when the two surfaces are uncorrelated *h* ~ *N* (*μ*_*zt*_ − *μ*_*zb*_*, s*_*zt*_^2^ + *s*_*zb*_^2^). The scale invariant features in our numerically generated fractures result from the fractal nature of the surface roughness^56^.

The aperture Gaussian distribution *f*_*h*_ allows us to obtain the lower bound of the *P*_*c*_-*S*_*w*_ curve: the pore volume distribution is *f*_*v*_ = (*h* × Δ*l*^2^)*f*_*h*_, then, the cumulative distribution function of *f*_*v*_ (from largest to smallest *h*) normalized by the integral of (*h* × Δ*l*^2^) corresponds to the wetting phase saturation *S*_*w*_ with capillary pressure *P*_*c*_ = *T*_*s*_/*h*.

## Conclusions

The capillarity pressure vs. saturation response of fractured rock masses is needed for long-term analyses. However, the time needed to reach thermodynamic equilibrium and size-dependent fracture topology limit our ability to experimentally gather relevant capillarity-saturation curves.

Numerical experiments show that the capillary pressure versus saturation *P*_*c*_-*S*_*w*_ curve for a fractured rock mass is determined by pore-scale characteristics in the fractures and the matrix, including aperture and pore size statistics, spatial variability and connectivity.

The fracture surface roughness can be synthesized as a sum of independent sinusoids. A power law relates the sinusoidal amplitudes to their wave wavelength. The central limit theory emerges and the aperture size follows a Gaussian distribution. The mean aperture increases with fracture-size, yet, the coefficient of variation *s*_*h*_/*μ*_*h*_ is scale independent.

While the wetting phase could remain occluded in the matrix during non-wetting fluid invasion, trapping in fractures is limited. Wetting fluid connectivity allows for the wetting fluid to escape through a network of connected corners formed by the surface roughness or via the permeable porous matrix. Long-term equilibrium in liquid–vapor systems further promotes non-trapping conditions in fractures.

The increase in normal stress contracts the aperture and results in higher capillary pressure, increased aperture variability, increased true contact area and steeper *P*_*c*_-*S*_*w*_ curves. Capillary invasion reflects a slight anisotropy during shear and affects saturation at low capillary pressures. The three-parameter van Genutchen model captures the fracture *P*_*c*_*-S*_*w*_ response and its evolution with normal stress and shear displacement. While the capillary entry value is inversely proportional to the fracture size, the normalized capillarity-saturation curves (*P*_*c*_*/P*_*0*_*)* − *S*_*w*_ follow similar trends and are scale independent.

At equilibrium, fracture spacing and aperture statistics combine with the matrix porosity to determine the capillary pressure versus saturation curve for a rock mass and its storativity. In the absence of gouge, the fracture *P*_*c*_-*S*_*w*_ curve controls the entry pressure, whereas the matrix regulates the rock mass residual saturation at high capillary pressures.

## Data Availability

The datasets used and/or analyzed during the current study are available from the corresponding author.

## List of symbols

*a*_*i*_ [m]: Sinusoid amplitude
*A*_*c*_ [m^2^]: True contact fracture area
*A*_*f*_ [m^2^]: Apparent fracture area
*C*: Fourier transform scaling factor
*d* [m]: Fracture spacing
*f*: Probability density function (sub-z_i_: sinusoid; sub-z: sum of the sinusoids; sub-h: aperture)
*G* [m^3^]: Power spectral density
*h* [m]: Fracture aperture (sub-avg: average)
*L* [m]: Fracture size (sub-x: x-direction; sub-y: y-direction)
*m*: Fitting parameters in the van Genuchten model (sub-1: first parameter; sub-2: second parameter)
*n*: Matrix porosity
*N*: Aperture field size
*N*_*p*_: Lattice size for invasion percolation model
*P*_*0*_ [Pa]: Fitting parameter in the van Genuchten model
*P*_*c*_ [Pa]: Capillary pressure
*r* [m]: Matrix pore size
*s* [m]: Standard deviation (sub-h: aperture; sub-z: surface topography; sub-zt: top surface, sub-zb: bottom surface)
*S*_*w*_: Wetting phase saturation (sup-RM: rock mass)
*T*_*s*_ [N/m]: Interfacial tension
*V*_*v*_ [m^3^]: Void volume (sup-F: fracture; sup-M: matrix)
*X*(*λ*) [m]: Asperity amplitude for a given wavelength
*z* [m]: Surface topography (sub-t: top surface; sub-b: bottom surface)
*α* [m^3^]: Spectral density at *λ* = 1 m
*β*: Power spectral density sensitivity to wavelength
*δ* [m]: Fracture displacement (sub-v: vertical; sub-x: x-direction)
*Δl* [m]: Cell size for invasion percolation model
*Δx* [m]: Sampling interval for fracture generation
*η*: Void fraction (sup-F: fracture; sup-M: matrix)
*θ* [deg]: Contact angle
*λ* [m]: Asperity wavelength (sub-max: maximum)
*μ* [m]: Mean (sub-h: aperture; sub-z: surface topography; sub-zt: top surface, sub-zb: bottom surface)
*σ* [Pa]: Normal stress
*σ*_*y*_ [Pa]: Material yield stress
*φ*_*i*_ [m]: Sinusoid phase
*ω*_*i*_ [m]: Sinusoid frequency
